# Divergent Rabies Virus Variant of Probable Bat Origin in 2 Gray Foxes, New Mexico, USA

**DOI:** 10.3201/eid2806.211718

**Published:** 2022-06

**Authors:** Rene E. Condori, Adam Aragon, Mike Breckenridge, Kendra Pesko, Kerry Mower, Paul Ettestad, Sandra Melman, Andres Velasco-Villa, Lillian A. Orciari, Pamela Yager, Daniel G. Streicker, Crystal M. Gigante, Clint Morgan, Ryan Wallace, Yu Li

**Affiliations:** Centers for Disease Control and Prevention, Atlanta, Georgia, USA (R.E. Condori, A. Velasco-Villa, L.A. Orciari, P. Yager, C.M. Gigante, C. Morgan, R. Wallace, Y. Li);; New Mexico Department of Health, Albuquerque, New Mexico, USA (A. Aragon, M. Breckenridge, K. Pesko, P. Ettestad, S. Melman);; New Mexico Department of Game and Fish, Santa Fe, New Mexico, USA (K. Mower);; University of Glasgow, Glasgow, Scotland, UK (D.G. Stricker)

**Keywords:** rabies, rabies virus, lyssavirus, viruses, divergent virus variant, bats, bat origin, gray foxes, phylogenetic analysis, spillover, zoonoses, New Mexico, United States

## Abstract

In the Western Hemisphere, bat-associated rabies viruses (RABVs) have established independent transmission cycles in multiple mammal hosts, forming genetically distinct lineages. In New Mexico, USA, skunks, bats, and gray foxes are rabies reservoir hosts and represent a public health risk because of encounters with humans. During 2015 and 2019, two previously undescribed RABVs were detected in 2 gray foxes (*Urocyon cinereoargenteus*) in Lincoln County, New Mexico. Phylogenetic analysis of the nucleoprotein gene indicated that the isolates are a novel RABV variant. These 2 cases probably represent repeated spillover events from an unknown bat reservoir to gray foxes. Molecular analysis of rabies cases across New Mexico identified that other cross-species transmission events were the result of viral variants previously known to be enzootic to New Mexico. Despite a robust rabies public health surveillance system in the United States, advances in testing and surveillance techniques continue to identify previously unrecognized zoonotic pathogens.

Rabies is a viral zoonotic disease that infects the central nervous system of mammals and causes a highly lethal acute encephalitis. *Rabies lyssavirus* is the most prevalent of the 17 recognized species of the genus *Lyssavirus* and is genetically grouped within the phylogroup I (*1*,*2*). Rabies virus (RABV) is distributed worldwide and has an estimated human rabies death toll of >59,000 annually. Most human rabies deaths are associated with dog-mediated rabies, predominantly in Asia, Africa, and several countries in the Western Hemisphere (*3*). Rabies is commonly transmitted through direct contact with the saliva of rabid animals; humans or any susceptible mammal usually become infected through a bite. After potential exposure to RABV, if postexposure prophylaxis (PEP) is not administered before symptom onset, the outcome will nearly always be fatal (*4*).

In the Western Hemisphere, 2 genetic lineages of RABV have been identified: Cosmopolitan Dog lineage and New World lineage. The Cosmopolitan Dog lineage was introduced during European colonization; dog-to-dog transmission and host switching to other terrestrial mesocarnivores enabled this lineage to spread and establish across the Americas and some Caribbean islands. The New World lineages circulate mainly within bat populations, with several exceptions of lineages that shifted to terrestrial mesocarnivores (*5*,*6*).

The United States recognized dog and wildlife rabies as a problem and organized large-scale public health efforts to control the disease as early as 1944 (https://wwwn.cdc.gov/nndss/conditions/rabies-animal), the year in which a consensus agreement was reached to consider rabies a reportable disease (*7*). Interrupting the chain of dog-to-dog transmission thorough immunization led to the milestone of eliminating rabies circulating in dogs (*8*). According to the most recent annual surveillance reports, since 2015, bats have become the most commonly reported rabies reservoir species in the continental United States (*9*,*10*). RABVs circulating in bat populations are incredibly diverse.

Monoclonal antibodies (mAbs) provide evidence of antigenic differences among RABVs, and mAbs patterns form the basis for determining conventional RABV variant nomenclature (*11*). However, RABV variant identification by using mAbs might not be able to provide appropriate resolution because of genetic variation, particularly when applied to the highly diverse bat RABV. Therefore, a comprehensive genetic analysis is frequently used to understand transmission dynamics and explore genetic differences (*12*). In the United States, RABV variants are often named on the basis of the presumptive reservoir host (e.g., *Tadarida brasiliensis* bats); >18 different recognized bat-specific variants have been identified (*13*,*14*). Detailed genetic studies have suggested several instances in which RABV circulating in bats has shifted to terrestrial mammals. Enzootic cycles of RABV from bat origin have been established by host shift events separately in raccoons (*Procyon lotor*) and skunks (*Mephitis mephitis*) (*15*).

Host shift events are rare, and the factors that lead to a successful host shift are poorly understood. Some studies have linked such events to ecologic, viral, or host factors that might contribute to long-term establishment (*16*–*18*). Circulation of novel RABV variants in wildlife species can remain unnoticed unless there is an outbreak or an event that leads to an infected animal reported the National Rabies Surveillance System (https://www.healthypeople.gov), in which testing and additional virus characterization can detect unexpected virus‒host infections (*19*,*20*). Laboratory-based surveillance using molecular tools is useful to identify genetic changes and explore relatedness at a more refined level, which can help to identify novel RABV variants (*21*).

New Mexico is known to have >3 RABV enzootic cycles represented by skunks (south-central skunk variant), gray foxes (Arizona gray fox variant), and numerous variants associated with bats. During 2000‒2020, the state surveillance system detected 275 rabies cases in wildlife species and 14 cases in domestic animals (https://nmhealth.org/about/erd/ideb/zdp/rab). In 2015, a woman in Lincoln County was attacked by a gray fox and appropriate PEP was given; a sample showed positive results for rabies, but preliminary antigenic and molecular analysis did not align with known RABV variants. In 2019, a second isolate collected from a gray fox that bit a man in the same county, but in a different city, showed a similar genetic pattern. The purpose of this study was to characterize these divergent RABV isolates from gray foxes in Lincoln County and investigate potential reservoir host species.

## Materials and Methods

### RABV Samples

All samples were collected as part of routine public health surveillance activities, and no animal sampling was performed for this study. Of the 289 samples tested that were positive for RABV in New Mexico during 2000–2020, a total of 90 were available for molecular characterization (Appendix 1 Table 1). Of the 90 samples, 58 were analyzed by the Scientific Laboratory Division Department of Health of New Mexico (SLD-NM) and 32 were submitted for rabies virus characterization to the National Rabies Reference Laboratory (NRRL), Division of Global Migration and Quarantine, National Center for Emerging and Zoonotic Infectious Diseases, Centers for Disease Control and Prevention (CDC) (Appendix 2 Table). Five isolates from gray foxes collected by the surveillance system in Arizona and 3 archived isolates from *Lasiurus intermedius* bats from Florida were also included. In addition, from the batch of isolates submitted to CDC, we tested isolates A15-0755 and A19-2238 from gray foxes collected in Lincoln County during 2015 and 2019 by using a panel of RABV nucleoprotein mAbs (*22*) and obtained the whole genomes.

### RNA Extraction and Reverse Transcription PCR Amplification

We extracted total RNA from brain tissue by using either TRIzol (Invitrogen, https://www.thermofisher.com) or the Direct-zol RNA MiniPrep Kit (Zymo Research, https://www.zymoresearch.com) according to the manufacturer’s instructions. We performed traditional reverse transcription PCR to produce partial and full nucleoprotein gene amplicons by using overlapping nucleoprotein gene primers described (*19*). To obtain the whole RABV genome for isolates A15-0755 and A19-2238, we synthesized cDNA by using specific primer LN34 forward (*23*) with avian myeloblastosis virus reverse transcriptase (Roche, Sigma-Aldrich, https://www.sigmaaldrich.com). PCR amplicons suitable to cover the entire genome were generated by using 6 overlapping pair of primers (Appendix 1 Table 2) and Takara long amplicon Taq polymerase (Takara Bio USA, https://www.takarabio.com).

### Nucleotide Sequencing and Phylogenetic Analysis

We obtained partial and complete nucleoprotein gene sequences by using overlapping primers with the BigDye Terminator v1.1 Cycle Sequencing Kit (Thermo Fisher). We sequenced the amplicons in a 3730 DNA Analyzer (Applied Biosystems, (Thermo Fisher) by using standard Sanger sequencing method (*19*). SADB119 (GenBank accession no. M31046) sequence was used as a reference to assemble the partial and full nucleoprotein gene and the whole genome. Nucleoprotein gene sequences were edited by using Bioedit software (*24*). We included high-throughput sequencing to obtain whole genome sequences for isolates A15-0755 and A19-2238. We generated amplicons >2 kb by using specific primers (Appendix 1 Table 2) and pooled and fragmented all amplicons for each isolate to 500 bp by using Covaris S220 (https://www.covaris.com).

We quantified DNA by using a Qubit instrument (Thermo Fisher), performed library preparation by using the Accel-NGS 2S plus DNA Library Kit (Swift Biosciences, https://www.idtdna.com), and obtained sequence reads in a MiSeq Instrument (Illumina, https://www.illumina.com). We assembled genomes by using CLC Genomics Workbench version 20 (https://digitalinsights.qiagen.com), trimmed reads with a quality limit of 0.05, then mapped to reference JQ685895 to generate a majority draft consensus. We generated final genomes by mapping reads back to draft genomes and extracting consensus sequences with minimum 50× read depth; we inserted ambiguous bases with a noise threshold of 10%. We submitted sequences generated in this study to GenBank (accession nos. OM202982‒3049).

We aligned sequences generated in this study by using ClustalW in Geneious 10.2.2 (https://www.geneious.com). We conducted phylogenetic analysis by using a Bayesian approach in the BEAST v1.10.4 package (*25*). To estimate the time since the most recent common ancestor of fox-associated viruses and other bat and carnivore-associated RABVs, we analyzed 141 nucleoprotein gene sequences (Appendix 1 Table 2). Preliminary analyses used iqTree to compare substitution models and obtain a maximum-likelihood topology without a molecular clock assumption (*26*).

We used the most likely substitution model according to the corrected Akaike Information (generalized time reversible plus finite sites plus invariant sites plus gamma 4) in subsequent analyses. We checked for temporal signal in our sequence data by correlating sampling time with root-to-tip divergence by applying TempEst (*27*) to the maximum-likelihood tree estimated in iqTree (*28*). We subsequently performed Bayesian phylogenetic analyses in duplicate by using the relaxed lognormal molecular clock and the Bayesian skyline demographic model with BEAST (*25*,*29*). We partitioned codon positions 1 and 2 separately from codon position 3 and applied the generalized time reversible plus invariant sites plus gamma substitution model to both partitions. We modeled 1 year of uncertainty around each sampling date. We performed each analysis for 100 million generations, sampling trees, and parameters every 5,000 steps and checked chains for convergence within and between runs in Tracer (https://beast.community/trac). We combined trees and log files in LogCombiner (https://beast.community/logcombiner) after removing 10 million generations as burn-in; we further thinned tree files to be sampled every 10,000 steps. This strategy led to effective sample size values >200 for all parameters. We visualized all the phylogenetic trees by using Fig Tree v1.4.0 (*30*).

**Figure 1:**
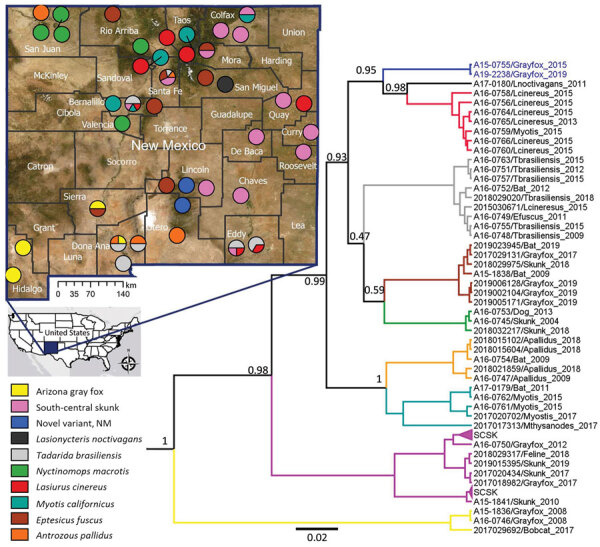
Phylogenetic tree based on partial nucleoprotein gene (348 bp) sequences and geographic distribution of rabies virus (RABV) variants, New Mexico, USA. The tree was constructed by using representative isolates and only posterior values leading to the RABV variants are included on the tree nodes. Three major clades were identified in New Mexico. The RABV variants are displayed in distinct colors in the tree and map according to the legend included in the figure. Blue indicates novel RABV isolates collected from gray fox in Lincoln County. Accuracy of the location in the map is at city level; for samples that did not have city information, the location was randomly assigned within the county. Numbers along branches are bootstrap values. Scale bar indicates nucleotide substitutions per site.

We calculated genetic distance in Geneious and visualized rabies distribution across New Mexico by exporting partial nucleoprotein gene Bayesian tree into ArcGIS desktop v10.7.1 (https://www.esri.com). We sourced administrative boundaries (Figure 1) from GADM version 4 (Database of Global Administrative Areas; www.gadm.org) and specific imagery from Maxar Technologies Inc., (https://www.maxar.com) accessed from ESRI World Imagery.

## Results

During 2015 and 2019, two persons in Lincoln County were bitten by rabid gray foxes. Initially, we compared the full nucleoprotein gene sequence of the 2015 isolate, A15-0755, with sequences available in GenBank. The most similar sequence was the RABV isolate collected in Canada from a *Myotis lucifugus* bat (GenBank accession no. AF351837) and characterized as silver-haired bat RABV variant (*Lasionycteris noctivagans* bats) with a sequence identity of 93.25%. A15-0755 showed an atypical reaction pattern with mAbs 2 and 11; a similar reaction pattern was observed in a historic isolate from a northern yellow bat (*Lasiurus intermedius)* collected in Florida and archived at CDC. During 2019, a second isolate, A19-2238, from a second rabid gray fox from the same county was identified in SLD-NM and was further characterized at CDC. A full nucleoprotein gene sequence showed a single nucleotide mismatch with the 2015 isolate. A19-2238 produced a Mab reaction pattern similar to a RABV variant circulating in hoary bats (*Lasiurus cinereus*)*.*

### Phylogenetic Analysis

Bayesian phylogenetic inference of partial and complete nucleoprotein genes in BEAST showed consistent tree topologies, grouping the New Mexico isolates into 3 major clades. The first clade of bat RABV variants included isolates from bats and terrestrial carnivores that were presumed to be the result of cross-species transmission (Figure 1). Within this clade, 7 previously known RABV genetic variants were identified. Isolates A15-0755 and A19-2238 formed an independent branch. Phylogenetic analysis did not identify a close relationship with any RABV sequences available in GenBank. Both isolates were most closely related to, but still highly divergent from (mean ± SD identity 95.85% ± 2.21%), the branches containing RABVs detected in *Lasiurus cinereus* bats and *L. noctivagans* bats. The third RABV isolate from a gray fox in Lincoln County (A15-1838) clustered within a branch that contained isolates from *Eptesicus fuscus* bats, which indicated a spillover event of a bat RABV variant to a terrestrial mammal.

We also identified other spillover events from bats to terrestrial mammals (Appendix 2 Table). The variant commonly circulating among *E. fuscus* bats was detected in gray foxes and skunks, and a RABV variant circulating in *Nycintomops macrotis* bats was found in a skunk isolate (A16-0745) from San Juan County and a dog isolate (A16-0753) from Valencia County. The second clade containing isolates identified as south-central skunk RABV variant included isolates collected from gray foxes, coyotes, and domestic cats. The third clade identified as Arizona gray fox contained 2 isolates from gray foxes and 2 from bobcats. Circulation of variants A16-0745 and A16-0753 in terrestrial mammals was consistent with previous reports (Figure 1) (*31*,*32*). Bat virus variants were found throughout the state but showed no notable epidemiologic pattern.

**Figure 2:**
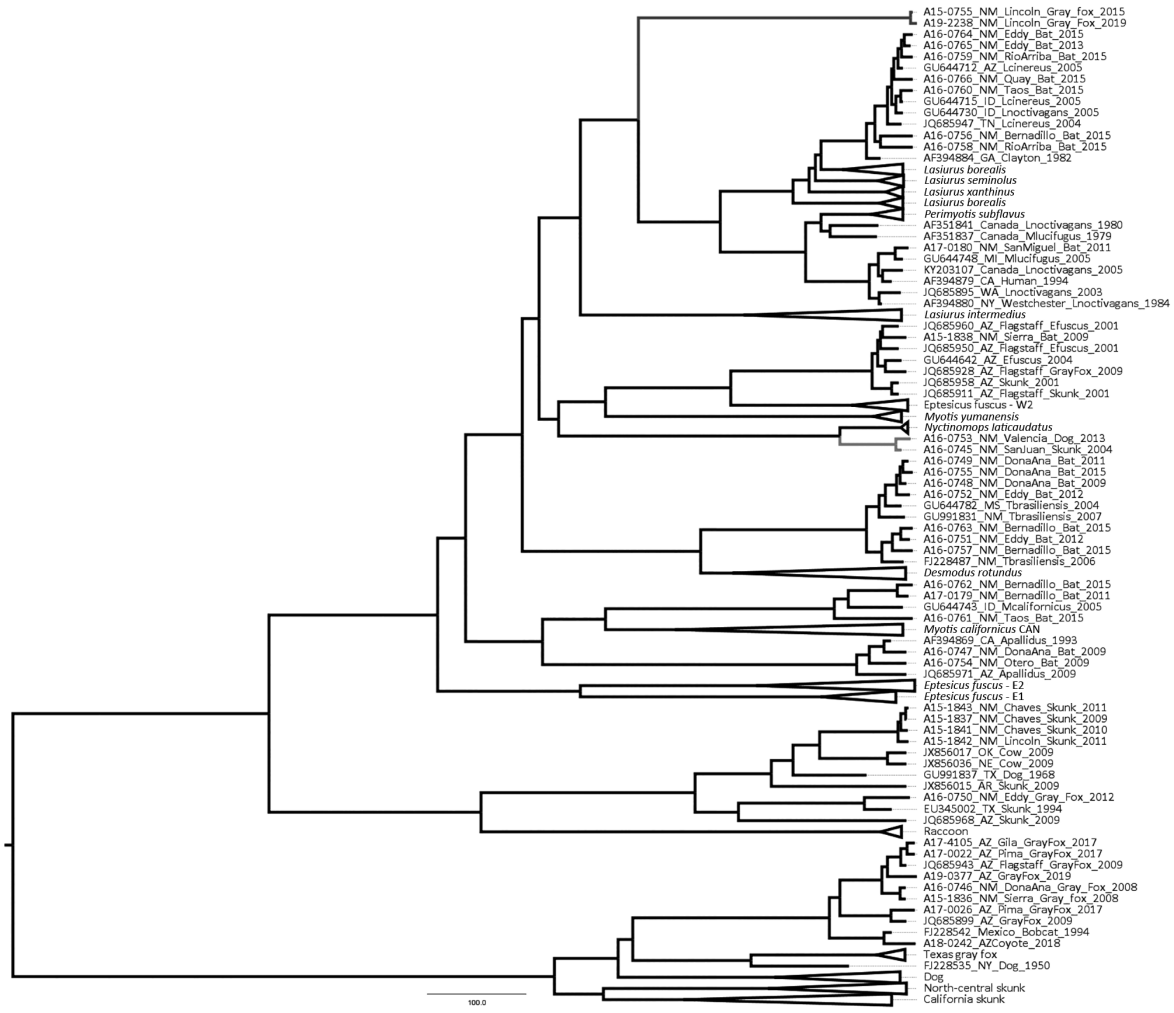
Maximum clade credibility tree using full nucleoprotein gene sequences of RABV variants identified in New Mexico, USA, and representative sequences from RABV variants in the Western Hemisphere. Values in the nodes indicate estimates for the posterior clade probability for each RABV variants. Branch in blue indicates novel RABV variant that includes the 2 isolates from Lincoln County, and branch in green indicates RABV associated with *Nyctinomops macrotis* bats. Scale bar indicates nucleotide substitutions per site. RABV, rabies virus.

Phylogenetic inferences including all 32 nucleoprotein gene sequences from New Mexico generated at CDC and representative sequences retrieved from GenBank produced a phylogenetic tree with similar topologies to the partial nucleoprotein gene (Figure 1). Gray fox isolates A15-0755 and A19-2238 formed a unique branch high posterior support (*1*); the most similar isolate available on GenBank (accession no. AF351837) clustered separately within the *Perimyotis subflavus* and *Lasionyteris noctivagans* bat RABV variants (Figure 2).

### Novel Gray Fox Isolates

The substitution rate along the branch leading to the 2 gray fox isolates identified as a novel RABV variant was 1.6 × 10^‒4^ substitutions/site/year (95% highest posterior density 9.2 × 10^‒5^–2.4 × 10^‒4^ substitutions/site/year). The substitution rate across the entire tree was 1.7 × 10^‒4^ substitutions/site/year (95% highest posterior density (1.2 × 10^‒4^–2.2 × 10^‒4^ substitutions/site/year), indicating a similar rate of evolution. The nucleoprotein gene genetic distance between RABV variants examined showed that the highest identity to isolate A15-0755 was 93.19% for isolate AF351837 (silver-haired bat variant)¸ and the identity for isolate A19-2238 was 93.11% (Appendix 1 Table 3). We conducted a BLAST (https://blast.ncbi.nlm.nih.gov/Blast.cgi) search of the glycoprotein gene from the 2 novel RABV variant isolates in GenBank; isolate KJ174682 from an *E. fuscus* bat and described as EF-W3 had the closest glycoprotein gene identity (90.42% nt). Nucleotide comparison across the entire genome of isolates A15-0755 and A19-2238 showed 99.78% identity.

The nucleoprotein gene, which is highly conserved and a target for laboratory diagnosis, showed a single synonymous substitution at nt position 333. The glycoprotein gene, which induces production of virus-neutralizing antibodies that provide immunologic protection, showed a nonsynonymous substitution at nt position 554, resulting in a substitution of the amino acid tyrosine at position 184 by serine (Y184S). Phylogenetic analysis using a reduced set of glycoprotein gene sequences placed isolates A15-0755 and A19-2238 in an independent branch, closely related to *L. noctivagans* and *P. subflavus* bats, that has well-supported posterior values (Appendix 2 Figure).

### Other Isolates

Two other isolates A16-0745 (skunk 2004) and A16-0753 (dog 2013) were compared with available sequences in GenBank. The partial nucleoprotein (244 bp) of a skunk isolate (A16-0745) showed 100% identity with RABV isolate AY960093 from GenBank, previously detected in an *N. macrotis* bat in Colorado, and 99.5% to isolate AY170304 from GenBank, detected in Arizona. The full nucleoprotein gene showed 98.0% (A16-0745) and 97.8% (A16-0753) identities with a RABV (GenBank accession no. KM594034) found in *N. laticaudatus* bats from Brazil. In the phylogenetic analysis using the full nucleoprotein gene, we found that both isolates branched independently and contained only these 2 terrestrial mammals (Figure 2).

### Cross-Species Transmission

Of the 49 bat clade samples that had sequencing results, 11 (22.4%) instances of bat-to-terrestrial cross species transmission were detected. Of 37 isolates with the south-central skunk RABV variant, 9 (24.3%) were cross-species transmissions to nonskunk species, and 2 (50%) of the 4 gray fox RABV variant isolates were cross-species transmissions. There were no instances of a terrestrial RABV variant found in a bat.

## Discussion

In the United States, 7 unique RABV variants are defined on the basis of unique mAbs patterns, each associated with a specific terrestrial host species in terrestrial mammals (arctic fox, gray fox, striped skunk, raccoon, and mongoose) (*13*). Based on patterns of the specific mAbs used, the standard panel can differentiate >15 RABV variants (*33*). Robust rabies surveillance systems and regular virus characterization are used to not only define the geographic distribution of variants but also detect RABV spillover into nonreservoir wildlife or domestic species that might reflect changes in rabies epidemiology and affect human or animal health. This study was conducted after the New Mexico Department of Health confirmed human RABV exposures to confirmed rabid foxes and pursued additional laboratory-based methods to determine if these cases represented a shift in the epidemiology of rabies in the state. As a result of this investigation, a novel RABV variant was identified. Detection of this novel variant in 2 gray foxes, separated by 5 years, probably represents independent cross-species transmission events from a cryptic transmission cycle among a species of bats.

Available data for the United States show that the distribution of terrestrial RABV variants is geographically delimited; in contrast, the distribution of bat RABV variants is broader, and these variants show an abundant diversity, each variant associated with specific species (*9*,*14*). For example, according to the National Rabies Surveillance System, during 2008‒2018, at least 17,700 rabid bats were detected in 39 different bat species, but 54.9% of rabid bats were not identified to species, and 63% did not undergo variant typing (antigenic or molecular) (*34*,*35*). Given these apparent gaps in characterizing rabid bats in the United States, it should be no surprise that novel RABV variants are still being discovered. As characterization methods and bat identification guides (*36*) become more accessible, it is expected that a wider diversity of bat RABV variants will be detected. Furthermore, public health surveillance is biased toward animals with human or domestic animal exposures, potentially masking detection of RABV variants in species not commonly found near inhabited areas.

RABV isolates from New Mexico included in this study were derived from bats and terrestrial mammals collected in different locations spanning over 20 years. The phylogenetic inferences (Figure 1) clearly show 3 major clades supported that have high posterior values. All isolates were closely associated with RABV variants already described in New Mexico (*14*,*37*), except for the 2 isolates from gray foxes collected during 2015 and 2019 in Lincoln County. An extensive analysis of full nucleoprotein and glycoprotein genes supported the uniqueness of the isolates as an unrecognized RABV variant. Nucleotide analysis of the glycoprotein gene of the novel RABV variant had a specific glycoprotein gene mutation that is located in the antigenic site II, which is involved in stimulating the antibody response (*38*). Although the current rabies vaccine is effective in protecting against all lyssaviruses from phylogroup I, monitoring the nucleotide mutation across the glycoprotein gene on emergent RABVs in domestic and wild animals might help to predict if the vaccine will still be effective against these new viruses (*39*). This single mutation is not believed to lead to an escape from current RABV biologics, as shown by the lack of development of rabies in the 2 persons exposed to this virus after they received PEP.

On the basis of phylogenetic analysis, we reason that the reservoir of this novel RABV variant is most likely a bat from the group commonly referred to as migratory tree bats, including the genera *Lasiurus* and *Lasionycteris*. The isolates identified probably represent repeated spillover events from a bat reservoir into gray foxes in Lincoln County. This hypothesis is supported by the low frequency of detection of the variant (2 cases in 5 years), which might be expected because bats and wild terrestrial mammals have limited contact with humans and other terrestrial mammals unless they are sick or injured (*40*). On the basis of available data and analysis, we cannot provide enough evidence to prove that this variant represents a host shift from bat RABV variants into gray foxes; however, the question will remain open until the reservoir is determined. After rabies was recognized in the gray fox during 2015, an active surveillance program was enacted in Lincoln County and surrounding counties; however, no rabid terrestrial animals were detected during this 6-month effort. To increase the robustness of this analysis, isolates from additional rabid foxes or bats either in Lincoln County or neighboring areas are clearly needed. In nature, bats inhabit diverse ecologic niches (*41*). Migratory tree bats usually travel long distances, which opens the possibility that this novel RABV variant might be present in other states or countries (*42*).

Although migratory tree bats are a major rabies reservoir in the United States, other species of migratory bats, such as *N. macrotis* (big free-tailed bats), commonly travel long distances, covering a range from South America to North America (*43*). Detecting rabies in *N. macrotis* bats is uncommon, and the availability of genetic data in GenBank is limited to 2 partial nucleoprotein gene sequences (300 bp): AY170304 (Arizona) and AY960093 (Colorado). The surveillance system in the United States detected 18 rabid *N. macrotis* bats during 2008‒2018; the highest incidences were in 2015 (n = 9) and 2010 (n = 4) (*10*,*34*,*44*–*46*). 

In this study, we found an *N. macrotis* bat RABV variant in a domestic dog and a skunk separated by 11 years. Analysis of the full nucleoprotein gene provided high posterior support that the RABV variant detected in United States shared a recent ancestor with a RABV variant found in Brazil in *N. laticaudatus* bats (*47*). The finding of a relatively rare RABV variant in a migratory bat species represents a reminder that RABVs can be carried long distances by reservoir species and could represent a method of introduction of exotic RABVs into the United States, yet another example of the need for adequate surveillance, routine species identification, and RABV characterization (*48*).

Antigenic characterization is useful to rapidly identify the common RABV variants in the United States (*49*). The antigenic patterns of the isolates from Lincoln County gave conflicting results; the isolates showed different patterns, despite having 99.9% genetic similarity. These discrepancies in the interpretation of the mAb results demonstrate the limitation of that method to differentiate RABVs within certain bat species. In comparison, the amplicon sequences generated by the LN34 assay (*50*), which target a highly conserved lead sequence of RABV genome, are able to confirm the distinct sequences for this novel RABV. This study highlights the need for RABV characterization when there are concerns about epidemiologic shifts to inform public health and animal health interventions. Despite extensive surveillance systems in the United States for RABV, virus characterization is not routinely performed. As genetic virus characterization becomes more routine, additional cryptic RABV transmission cycles probably will be recognized.

Appendix 1 Animals, primers, and nucleotide identities used for study of divergent rabies virus variant of probable bat origin in 2 gray foxes, New Mexico, USA.

Appendix 2 Additional information on divergent rabies virus variant of probable bat origin in 2 gray foxes, New Mexico, USA.
