# Supplementary material for: Divergent Rabies Virus Variant of Probable Bat Origin in 2 Gray Foxes, New Mexico, USA
Source: Emerg Infect Dis. 2022 Jun;28(6):1137–45. doi: 10.3201/eid2806.211718 (PMC9155866; doi:10.3201/eid2806.211718)
Supplement: Appendix 2 — Additional information on divergent rabies virus variant of probable bat origin in 2 gray foxes, New Mexico, USA. [file 21-1718-Techapp-s2.pdf]

Article DOI: https://doi.org/10.3201/eid2806.211718

Divergent Rabies Virus Variant of Probable Bat Origin in 2 Gray Foxes, New Mexico, USA

Appendix 2 Table. Isolates sequenced this study and reference sequences used for the analysis*

Isolate	Country	State	County	City	Host source	Year	GenBank accession no.	RABV lineage	Reference
A15-0755; 2015012162	USA	New Mexico	Lincoln	Alto	*Urocyon cinereoargenteus*	2015	OM202983	Novel New Mexico	This study
A15-1836; 2008000832	USA	New Mexico	Sierra		*Urocyon cinereoargenteus*	2008	OM202984	Cosmopolitan (AZFX)	This study
A15-1837; 2009000595	USA	New Mexico	Chaves		*Mephitis mephitis*	2009	OM202985	South-central skunk	This study
A15-1838; 2009001979	USA	New Mexico	Sierra		Bat	2009	OM202986	*Eptesicus fuscus*-W1	This study
A15-1841; 2010039542	USA	New Mexico	Chaves		*Mephitis mephitis*	2010	OM202987	South-central skunk	This study
A15-1842; 2011021732	USA	New Mexico	Lincoln		*Mephitis mephitis*	2011	OM202988	South-central skunk	This study
A15-1843; 2011030508	USA	New Mexico	Chaves		*Mephitis mephitis*	2011	OM202989	South-central skunk	This study
A16-0745; 2004003088	USA	New Mexico	San Juan		*Mephitis mephitis*	2004	OM202990	*Nyctinomops macrotis*	This study
A16-0746; 2008003514	USA	New Mexico	Dona Ana		*Urocyon cinereoargenteus*	2008	OM202991	Cosmopolitan (AZFX)	This study
A16-0747; 2009002276	USA	New Mexico	Dona Ana		*Antrozous pallidus*	2009	OM202992	*Antrozous pallidus*	This study
A16-0748; 2009002549	USA	New Mexico	Dona Ana		*Tadarida brasiliensis*	2009	OM202993	*Tadarida brasiliensis*	This study
A16-0749; 2011031485	USA	New Mexico	Dona Ana		*Eptesicus fuscus*	2011	OM202994	*Tadarida brasiliensis*	This study
A16-0750; 2012026146	USA	New Mexico	Eddy		*Urocyon cinereoargenteus*	2012	OM202995	South-central skunk	This study
A16-0751; 2012034852	USA	New Mexico	Eddy		*Tadarida brasiliensis*	2012	OM202996	*Tadarida brasiliensis*	This study
A16-0752; 2012036136	USA	New Mexico	Eddy		Bat	2012	OM202997	*Tadarida brasiliensis*	This study
A16-0753; 2013038564	USA	New Mexico	Valencia		*Canis familiaris*	2013	OM202998	*Nyctinomops macrotis*	This study
A16-0754; 2009002942	USA	New Mexico	Otero		Bat	2009	OM202999	*Antrozous pallidus*	This study
A16-0755; 2015013805	USA	New Mexico	Dona Ana	La Mesa	*Tadarida brasiliensis*	2015	OM203000	*Tadarida brasiliensis*	This study
A16-0756; 2015016381	USA	New Mexico	Bernalillo	Albuquerque	*Lasiurus cinereus*	2015	OM203001	*Lasiurus cinereus*	This study
A16-0757; 2015018259	USA	New Mexico	Bernalillo	Albuquerque	*Tadarida brasiliensis*	2015	OM203002	*Tadarida brasiliensis*	This study
A16-0758; 2015018808	USA	New Mexico	Rio Arriba	Dixon	*Lasiurus cinereus*	2015	OM203003	*Lasiurus cinereus*	This study
A16-0759; 2015020795	USA	New Mexico	Rio Arriba	Espanola	*Myotis* sp	2015	OM203004	*Lasiurus cinereus*	This study
A16-0760; 2015024056	USA	New Mexico	Taos	Taos	*Lasiurus cinereus*	2015	OM203005	*Lasiurus cinereus*	This study
A16-0761; 2015025510	USA	New Mexico	Taos	El Prado	*Myotis* sp	2015	OM203006	*Myotis californicus*	This study
A16-0762; 2015027394	USA	New Mexico	Bernalillo	Albuquerque	*Myotis* sp	2015	OM203007	*Myotis californicus*	This study
A16-0763; 2015028251	USA	New Mexico	Bernalillo	Albuquerque	*Tadarida brasiliensis*	2015	OM203008	*Tadarida brasiliensis*	This study
A16-0764; 2015031004	USA	New Mexico	Eddy	Carlsbad	*Lasiurus cinereus*	2015	OM203009	*Lasiurus cinereus*	This study
A16-0765; 2013021240	USA	New Mexico	Eddy		*Lasiurus cinereus*	2013	OM203010	*Lasiurus cinereus*	This study
A16-0766; 2015019329	USA	New Mexico	Quay	San Jon	*Lasiurus cinereus*	2015	OM203011	*Lasiurus cinereus*	This study
A17-0179; 2011031282	USA	New Mexico	Bernalillo		Bat	2011	OM203012	*Myotis californicus*	This study
A17-0180; 2011034775	USA	New Mexico	San Miguel		*Lasionycteris noctivagans*	2011	OM203013	*Lasionycteris noctivagans*	This study
A19-2238; 2019027361	USA	New Mexico	Lincoln	Capitan	*Urocyon cinereoargenteus*	2019	OM202982	Novel New Mexico	This study
2015030671	USA	New Mexico	Eddy	Carlsbad	*Bassariscus astutus*	2015	OM203014	*Tadarida brasiliensis*	This study
2016005216	USA	New Mexico	De Baca	Fort Sumner	*Mephitis*	2016	*	South-central skunk	This study
2016008511	USA	New Mexico	De Baca	Fort Sumner	*Mephitis mephitis*	2016	OM203015	South-central skunk	This study
2016022033	USA	New Mexico	De Baca	Fort Sumner	*Mephitis mephitis*	2016	OM203016	South-central skunk	This study
2016023718	USA	New Mexico	De Baca	Fort Sumner	*Mephitis mephitis*	2016	*	South-central skunk	This study
2017017313	USA	New Mexico	Colfax	Raton	*Myotis thysanodes*	2017	OM203017	*Myotis californicus*	This study
2017017380	USA	New Mexico	Dona Ana	Las Cruces	*Tadarida brasiliensis*	2017	*	*Tadarida brasiliensis*	This study
2017018982	USA	New Mexico	Roosevelt	Portales	*Urocyon cinereoargenteus*	2017	OM203018	South-central skunk	This study
2017020232	USA	New Mexico	Santa Fe	Edgewood	*Eptesicus fuscus*	2017	*	*Eptesicus fuscus*-W1	This study
2017020434	USA	New Mexico	Curry	Clovis	*Mephitis mephitis*	2017	OM203019	South-central skunk	This study
2017020702	USA	New Mexico	Santa Fe	Espanola	*Myotis* sp	2017	OM203020	*Myotis californicus*	This study
2017025341	USA	New Mexico	Colfax	Raton	*Mephitis mephitis*	2017	*	South-central skunk	This study
2017027177	USA	New Mexico	Santa Fe	Santa Fe	*Tadarida brasiliensis*	2017	*	*Tadarida brasiliensis*	This study
2017027736	USA	New Mexico	Hidalgo	Lordsburg	*Lynx rufus*	2017	*	Cosmopolitan (AZFX)	This study

Isolate	Country	State	County	City	Host source	Year	GenBank accession no.	RABV lineage	Reference
2017029131	USA	New Mexico	Lincoln	Ruidoso	*Urocyon cinereoargenteus*	2017	OM203021	*Eptesicus fuscus*-W1	This study
2017029692	USA	New Mexico	Hidalgo	Rodeo	*Lynx rufus*	2017	OM203022	Cosmopolitan (AZFX)	This study
2017034789	USA	New Mexico	Colfax	Maxwell	*Canis latrans*	2017	OM203023	South-central skunk	This study
2018001861	USA	New Mexico	Santa Fe	Santa Fe	*Mephitis mephitis*	2018	OM203024	South-central skunk	This study
2018011578	USA	New Mexico	Colfax	Cimarron	*Mephitis mephitis*	2018	OM203025	South-central skunk	This study
2018015102	USA	New Mexico	Santa Fe	Santa Fe	*Antrozous pallidus*	2018	OM203026	*Antrozous pallidus*	This study
2018015202	USA	New Mexico	Santa Fe	Santa Fe	*Mephitis mephitis*	2018	*	South-central skunk	This study
2018015604	USA	New Mexico	San Juan	Farmington	*Antrozous pallidus*	2018	OM203027	*Antrozous pallidus*	This study
2018018118	USA	New Mexico	De Baca	Fort Sumner	*Mephitis mephitis*	2018	OM203028	South-central skunk	This study
2018018773	USA	New Mexico	Bernalillo	Albuquerque	*Myotis lucifugus*	2018	*	*Tadarida brasiliensis*	This study
2018019209	USA	New Mexico	Santa Fe	Santa Fe	*Urocyon cinereoargenteus*	2018	OM203029	South-central skunk	This study
2018021859	USA	New Mexico	Dona Ana	Las Cruces	*Antrozous pallidus*	2018	OM203030	*Antrozous pallidus*	This study
2018026719	USA	New Mexico	San Juan	Aztec	*Nyctinomops macrotis*	2018	*	*Nyctinomops macrotis*	This study
2018028298	USA	New Mexico	Santa Fe	Santa Fe	*Mephitis mephitis*	2018	OM203031	South-central skunk	This study
2018029020	USA	New Mexico	Eddy	Carlsbad	*Tadarida brasiliensis*	2018	OM203032	*Tadarida brasiliensis*	This study
2018029317	USA	New Mexico	Curry	Clovis	*Felis catus*	2018	OM203033	South-central skunk	This study
2018029975	USA	New Mexico	Rio Arriba	Dulce	*Mephitis mephitis*	2018	OM203034	*Eptesicus fuscus*-W1	This study
2018032217	USA	New Mexico	San Juan	Bloomfield	*Mephitis mephitis*	2018	OM203035	*Nyctinomops macrotis*	This study
2019002104	USA	New Mexico	Santa Fe	Santa Fe	*Urocyon cinereoargenteus*	2019	OM203036	*Eptesicus fuscus*-W1	This study
2019005171	USA	New Mexico	Santa Fe	Santa Fe	*Urocyon cinereoargenteus*	2019	OM203037	*Eptesicus fuscus*-W1	This study
2019006128	USA	New Mexico	Santa Fe	Santa Fe	*Urocyon cinereoargenteus*	2019	OM203038	*Eptesicus fuscus*-W1	This study
2019007630	USA	New Mexico	Santa Fe	Santa Fe	*Mephitis mephitis*	2019	*	South-central skunk	This study
2019009072	USA	New Mexico	Santa Fe	Santa Fe	*Mephitis mephitis*	2019	*	South-central skunk	This study
2019009563	USA	New Mexico	De Baca	Fort Sumner	*Mephitis mephitis*	2019	*	South-central skunk	This study
2019009681	USA	New Mexico	De Baca	Fort Sumner	*Mephitis mephitis*	2019	*	South-central skunk	This study
2019010065	USA	New Mexico	Quay	Tucumcari	*Mephitis mephitis*	2019	*	South-central skunk	This study
2019011895	USA	New Mexico	Bernalillo	Albuquerque	*Mephitis mephitis*	2019	*	South-central skunk	This study
2019012604	USA	New Mexico	De Baca	Fort Sumner	*Urocyon cinereoargenteus*	2019	OM203039	South-central skunk	This study
2019013575	USA	New Mexico	De Baca	Fort Sumner	*Procyon lotor*	2019	*	South-central skunk	This study
2019014195	USA	New Mexico	Curry	Clovis	*Urocyon cinereoargenteus*	2019	*	South-central skunk	This study
2019015395	USA	New Mexico	Curry	Clovis	*Mephitis mephitis*	2019	OM203040	South-central skunk	This study
2019016184	USA	New Mexico	Roosevelt	Portales	*Mephitis mephitis*	2019	*	South-central skunk	This study
2019017081	USA	New Mexico	Curry	Clovis	*Mephitis mephitis*	2019	*	South-central skunk	This study
2019019506	USA	New Mexico	Colfax	Angel Fire	*Eptesicus fuscus*	2019	*	*Eptesicus fuscus*-W1	This study
2019020776	USA	New Mexico	Curry	Clovis	*Mephitis mephitis*	2019	*	South-central skunk	This study
2019022292	USA	New Mexico	Roosevelt	Portales	*Mephitis mephitis*	2019	*	South-central skunk	This study
2019022293	USA	New Mexico	Roosevelt	Portales	*Felis catus*	2019	*	South-central skunk	This study
2019023945	USA	New Mexico	Santa Fe	Santa Fe	Bat	2019	OM203041	*Eptesicus fuscus*-W1	This study
2019024119	USA	New Mexico	Santa Fe	Santa Fe	*Antrozous pallidus*	2019	*	*Antrozous pallidus*	This study
2019025160	USA	New Mexico	San Miguel	Las Vegas	Bat	2019	*	*Eptesicus fuscus*-W1	This study
2019026915	USA	New Mexico	Colfax	Angel Fire	*Mephitis mephitis*	2019	*	South-central skunk	This study
2019027778	USA	New Mexico	Rio Arriba	Abiquiu	*Nyctinomops macrotis*	2019	*	*Nyctinomops macrotis*	This study
2019027923	USA	New Mexico	Santa Fe	Santa Fe	*Urocyon cinereoargenteus*	2019	*	*Tadarida brasiliensis*	This study
2019033570	USA	New Mexico	Curry	Clovis	*Mephitis mephitis*	2019	*	South-central skunk	This study
SM870	USA	Florida			*Lasiurus intermedius*	1988	OM203047	*Lasiurus intermedius*	This study
SM871	USA	Florida			*Lasiurus intermedius*	1988	OM203048	*Lasiurus intermedius*	This study
SM873	USA	Florida			*Lasiurus intermedius*	1988	OM203049	*Lasiurus intermedius*	This study
A17-0022	USA	Arizona	Pima		*Urocyon cinereoargenteus*	2017	OM203042	Cosmopolitan (AZFX)	This study
A17-0026	USA	Arizona	Pima		*Urocyon cinereoargenteus*	2017	OM203043	Cosmopolitan (AZFX)	This study
A17-4105	USA	Arizona	Gila		*Urocyon cinereoargenteus*	2017	OM203044	Cosmopolitan (AZFX)	This study
A18-0242	USA	Arizona			*Canis latrans*	2018	OM203045	Cosmopolitan (AZFX)	This study
A19-0377	USA	Arizona			*Urocyon cinereoargenteus*	2019	OM203046	Cosmopolitan (AZFX)	This study

Isolate	Country	State	County	City	Host source	Year	GenBank accession no.	RABV lineage	Reference
EF31; 89RABL1461	Canada	Saskatchewan			*Eptesicus fuscus*	1989	AF351831	*Eptesicus fuscus* - E2	Nadin-Davis,S.A., et al 2001
ML7; 79RABL1020	Canada	Alta			*Myotis lucifugus*	1979	AF351837	*Lasionycteris noctivagans*	Nadin-Davis,S.A., et al 2001
LAN13; 80RABN4398	Canada	Ontario			*Lasionycteris noctivagans*	1980	AF351841	*Lasionycteris noctivagans*	Nadin-Davis,S.A., et al 2001
EF72; V570	USA	Connecticut			*Eptesicus fuscus*	1998	AF351854	*Eptesicus fuscus* - E1	Nadin-Davis,S.A., et al 2001
EF71; V569	USA	Connecticut			*Eptesicus fuscus*	1998	AF351860	*Eptesicus fuscus* - E1	Nadin-Davis,S.A., et al 2001
EF3; 72R4744	Canada	Ontario			*Eptesicus fuscus*	1972	AF351861	*Eptesicus fuscus* - E2	Nadin-Davis,S.A., et al 2001
2253	USA	California			*Antrozous pallidus*	1993	AF394869	*Antrozous pallidus*	Rodhe, R.E. et al, 2004
1566	USA	California	Pluma		*Myotis californicus*	1987	AF394871	*Myotis californicus*	Rohde,R.E., et al 2004
872	USA	Florida	Sarasota		*Lasiurus intermedius*	1988	AF394878	*Lasiurus intermedius*	Rodhe, R.E. et al, 2004
2247	USA	California			*Homo sapiens*	1994	AF394879	*Lasionycteris noctivagans*	Rodhe, R.E. et al, 2004
2152	USA	New York	Westchester		*Lasionycteris noctivagans*	1984	AF394880	*Lasionycteris noctivagans*	Rodhe, R.E. et al, 2004
1435	USA	Arkansas	Houston		*Pipistrellus subflavus*	1991	AF394881	*Perimyotis subflavus*	Rodhe, R.E. et al, 2004
2698	USA	Tennessee			*Homo sapiens*	1994	AF394882	*Perimyotis subflavus*	Rodhe, R.E. et al, 2004
446	USA	Georgia	Clayton		*Lasiurus cinereus*	1982	AF394884	*Lasiurus cinereus*	Rodhe, R.E. et al, 2004
885	USA	Florida	Escambia		*Lasiurus borealis*	1988	AF394885	*Lasiurus borealis*	Rodhe, R.E. et al, 2004
2085	USA	Texas	Walker		*Lasiurus borealis*	1986	AF394886	*Lasiurus borealis*	Rodhe, R.E. et al, 2004
804	USA	California	Lake		*Eptesicus fuscus*	1987	AF394887	*Eptesicus fuscus*-W2	Rodhe, R.E. et al, 2004
136	USA	Pennsylvania	Perry		*Eptesicus fuscus*	1984	AY039226	*Eptesicus fuscus* - E1	Rodhe, R.E. et al, 2004
2049	USA	Colorado	El Paso		*Eptesicus fuscus*	1985	AY039228	*Eptesicus fuscus* - E2	Rohde,R.E., et al 2004
132	USA	Pennsylvania	Adams		*Eptesicus fuscus*	1984	AY039229	*Eptesicus fuscus* - E2	Rohde,R.E., et al 2004
3659	USA	Arizona	Flagstaff		*Nyctinomops macrotis*	1997	AY170304	*Nyctinomops macrotis*	Leslie,M.J., et al 2006
SHBRV-18	USA					NA	AY705373	*Lasionycteris noctivagans*	Faber,M., et al 2004
conm5293	USA	Colorado			*Nyctinomops macrotis*	NA	AY960093	*Nyctinomops macrotis*	Shankar,V., et al 2005
A033756NYrac1584	USA	New York	Westchester		*Procyon lotor*	2003	DQ886041	Eastern raccoon	Biek,R., et al, 2007
PA23	USA	Pennsylvania	Fulton		*Procyon lotor*	2004	EF508139	Eastern raccoon	Biek,R., et al, 2007
V211	USA	Texas			*Mephitis mephitis*	1994	EU345002	South-central skunk	Szanto,A., et al, 2007, un[ub. data
NewMexicoDG2006	USA	New Mexico			*Canis familiaris*	2006	FJ228487	*Tadarida brasiliensis*	Velasco-Villa, A., et al 2008
2153Salvhm02	USA				*Homo sapiens*	2002	FJ228492	*Desmodus rotundus*	Velasco-Villa, A., et al 2008
3901Mxmichbv03	Mexico	Michoacan			*Bos taurus*	2003	FJ228493	*Desmodus rotundus*	Velasco-Villa, A., et al 2008
412Mxyucdg02	Mexico	Yucatan			*Canis familiaris*	2002	FJ228525	Cosmopolitan (Dog)	Velasco-Villa, A., et al 2008
FLdg1994	USA	Florida			*Canis familiaris*	1994	FJ228528	Cosmopolitan (Dog)	Velasco-Villa, A., et al 2008
H2USTXWillcy98	USA	Texas			*Canis latrans*	1998	FJ228530	Cosmopolitan (Dog)	Velasco-Villa, A., et al 2008
DgNYKprwsky1950	USA	New York			*Canis familiaris*	1950	FJ228535	Cosmopolitan (Dog)	Velasco-Villa, A., et al 2008
TX692007	USA	Texas			*Canis latrans*	2007	FJ228536	Cosmopolitan (TXFX)	Velasco-Villa, A., et al 2008
TX672007	USA	Texas			*Urocyon cinereoargenteus*	2007	FJ228538	Cosmopolitan (TXFX)	Velasco-Villa, A., et al 2008
3148Mxchihbct94	Mexico	Chihuahua			*Lynx rufus*	1994	FJ228542	Cosmopolitan (AZFX)	Velasco-Villa, A., et al 2008
AZ1968	USA	Arizona			*Eptesicus fuscus*	2004	GU644642	*Eptesicus fuscus*-W1	Stricker, D.G., et al 2010
AZ7590	USA	Arizona			*Eptesicus fuscus*	2005	GU644643	*Lasiurus xanthinus*	Stricker, D.G., et al 2010
CA29	USA	California			*Eptesicus fuscus*	2002	GU644645	*Eptesicus fuscus*-W2	Stricker, D.G., et al 2010
IA381	USA	Iowa			*Eptesicus fuscus*	2005	GU644655	*Eptesicus fuscus* - E2	Stricker, D.G., et al 2010
MI1865	USA	Michigan			*Eptesicus fuscus*	2005	GU644667	*Eptesicus fuscus* - E2	Stricker, D.G., et al 2010
WA1833	USA	Washington			*Eptesicus fuscus*	2005	GU644693	*Eptesicus fuscus*-W2	Stricker, D.G., et al 2010
WA2017	USA	Washington			*Eptesicus fuscus*	2005	GU644695	*Eptesicus fuscus* - E2	Stricker, D.G., et al 2010
FL701	USA	Florida			*Lasiurus borealis*	2003	GU644698	*Lasiurus seminolus*	Stricker, D.G., et al 2010
TN33	USA	Tennessee			*Lasiurus borealis*	2004	GU644705	*Lasiurus borealis*	Stricker, D.G., et al 2010
TX6070	USA	Texas			*Lasiurus borealis*	2003	GU644707	*Lasiurus borealis*	Stricker, D.G., et al 2010
TX5276	USA	Texas			*Lasiurus borealis*	2004	GU644709	*Lasiurus borealis*	Stricker, D.G., et al 2010
AZ1838	USA	Arizona			*Lasiurus cinereus*	2005	GU644712	*Lasiurus cinereus*	Stricker, D.G., et al 2010

Isolate	Country	State	County	City	Host source	Year	GenBank accession no.	RABV lineage	Reference
ID7227	USA	Idaho			*Lasiurus cinereus*	2005	GU644715	*Lasiurus cinereus*	Stricker, D.G., et al 2010
FL1024	USA	Florida			*Lasiurus intermedius*	2001	GU644722	*Lasiurus intermedius*	Stricker, D.G., et al 2010
FL905	USA	Florida			*Lasiurus intermedius*	2001	GU644724	*Lasiurus intermedius*	Stricker, D.G., et al 2010
FL978	USA	Florida			*Lasiurus intermedius*	2001	GU644725	*Lasiurus intermedius*	Stricker, D.G., et al 2010
FL1165	USA	Florida			*Lasiurus intermedius*	2004	GU644726	*Lasiurus intermedius*	Stricker, D.G., et al 2010
TX5433	USA	Texas			*Lasiurus intermedius*	2003	GU644728	*Lasiurus xanthinus*	Stricker, D.G., et al 2010
ID7376	USA	Idaho			*Lasionycteris noctivagans*	2005	GU644730	*Lasiurus cinereus*	Stricker, D.G., et al 2010
GA7034	USA	Georgia			*Lasiurus seminolus*	2003	GU644732	*Lasiurus seminolus*	Stricker, D.G., et al 2010
TX5850	USA	Texas			*Lasiurus seminolus*	2002	GU644736	*Lasiurus borealis*	Stricker, D.G., et al 2010
TX6127	USA	Texas			*Lasiurus seminolus*	2003	GU644737	*Lasiurus borealis*	Stricker, D.G., et al 2010
CA06	USA	California			*Lasiurus xanthinus*	2004	GU644740	*Lasiurus xanthinus*	Stricker, D.G., et al 2010
ID7198	USA	Idaho			*Myotis californicus*	2005	GU644743	*Myotis californicus*	Stricker, D.G., et al 2010
WA1502	USA	Washington			*Myotis californicus*	2004	GU644745	*Myotis californicus*	Stricker, D.G., et al 2010
MI1100	USA	Michigan			*Myotis lucifugus*	2005	GU644748	*Lasionycteris noctivagans*	Stricker, D.G., et al 2010
AZ2857	USA	Arizona			*Myotis yumanensis*	2004	GU644751	*Myotis yumanensis*	Stricker, D.G., et al 2010
CA828	USA	California			*Myotis yumanensis*	2004	GU644752	*Myotis yumanensis*	Stricker, D.G., et al 2010
IN1657	USA	Indiana			*Pipistrellus subflavus*	2004	GU644757	*Perimyotis subflavus*	Stricker, D.G., et al 2010
TX5168	USA	Texas			*Pipistrellus subflavus*	2004	GU644758	*Perimyotis subflavus*	Stricker, D.G., et al 2010
MS076	USA	Mississippi			*Tadarida brasiliensis*	2004	GU644782	*Tadarida brasiliensis*	Stricker, D.G., et al 2010
NMBAT2007	USA	New Mexico			*Tadarida brasiliensis*	2007	GU991831	*Tadarida brasiliensis*	Velasco-Villa,A. and Rupprecht, C.E, 2016 unpub. data
TXdg1968	USA	Texas			*Canis familiaris*	1968	GU991837	South-central skunk	Velasco-Villa,A. and Rupprecht, C.E, 2016, unpub. data
H01/08	Colombia				*Homo sapiens*	2008	JF693456	*Desmodus rotundus*	Caicedo,Y., et al 2015
WA1185	USA	Washington			*Lasionycteris noctivagans*	2003	JQ595315	*Lasionycteris noctivagans*	Streicker, D.G., et al 2012
WA1066	USA	Washington			*Lasionycteris noctivagans*	2003	JQ595316	*Lasionycteris noctivagans*	Streicker, D.G., et al 2012
CA982	USA	California			*Mephitis mephitis*	1994	JQ685894	Cosmopolitan (CASK)	Kuzmin,I.V., et al 2012
WA1185	USA	Washington			*Lasionycteris noctivagans*	2003	JQ685895	*Lasionycteris noctivagans*	Kuzmin,I.V., et al 2012
SM5442	USA	Arizona	Flagstaff		*Eptesicus fuscus*	2001	JQ685897	*Eptesicus fuscus*-W1	Kuzmin,I.V., et al 2012
A10-0515	USA	Arizona			*Urocyon cinereoargenteus*	2009	JQ685899	Cosmopolitan (AZFX)	Kuzmin,I.V., et al 2012
FL769	USA	Florida			*Lasiurus seminolus*	2003	JQ685900	*Lasiurus seminolus*	Kuzmin,I.V., et al 2012
TN209	USA	Tennessee			*Lasiurus borealis*	2005	JQ685902	*Lasiurus borealis*	Kuzmin,I.V., et al 2012
CA04148	USA	California			*Eptesicus fuscus*	2004	JQ685903	*Eptesicus fuscus*-W2	Kuzmin,I.V., et al 2012
CA100	USA	California			*Eptesicus fuscus*	2005	JQ685909	*Eptesicus fuscus*-W1	Kuzmin,I.V., et al 2012
TX5960	USA	Texas			*Lasiurus xanthinus*	2002	JQ685910	*Lasiurus xanthinus*	Kuzmin,I.V., et al 2012
SM5077	USA	Arizona	Flagstaff		*Mephitis mephitis*	2001	JQ685911	*Eptesicus fuscus*-W1	Kuzmin,I.V., et al 2012
TX4904	USA	Texas			*Lasiurus intermedius*	2002	JQ685915	*Lasiurus intermedius*	Kuzmin,I.V., et al 2012
FL1010	USA	Florida			*Lasiurus intermedius*	2002	JQ685916	*Lasiurus intermedius*	Kuzmin,I.V., et al 2012
CO-Coyot-2010	USA	Colorado			*Eptesicus fuscus*	2010	JQ685917	*Eptesicus fuscus*-W2	Kuzmin,I.V., et al 2012
NJ2262	USA	New Jersey			*Lasiurus borealis*	2005	JQ685919	*Lasiurus borealis*	Kuzmin,I.V., et al 2012
EF	USA	Pennsylvania			*Eptesicus fuscus*	1984	JQ685920	*Eptesicus fuscus* - E1	Kuzmin,I.V., et al 2012
TN186	USA	Tennessee			*Pipistrellus subflavus*	2005	JQ685922	*Perimyotis subflavus*	Kuzmin,I.V., et al 2012
WAEF03	USA	Washington			*Eptesicus fuscus*	2004	JQ685925	*Eptesicus fuscus* - E2	Kuzmin,I.V., et al 2012
2403	USA	Arizona	Flagstaff		*Urocyon cinereoargenteus*	2009	JQ685928	*Eptesicus fuscus*-W1	Kuzmin,I.V., et al 2012
WA0173	USA	Washington			*Eptesicus fuscus*	2000	JQ685931	*Eptesicus fuscus*-W2	Kuzmin,I.V., et al 2012
A10-0514	USA				*Mephitis mephitis*	2009	JQ685938	South-central skunk	Kuzmin,I.V., et al 2012
AZBAT65094	USA	Arizona			*Eptesicus fuscus*	1981	JQ685942	*Eptesicus fuscus*-W2	Kuzmin,I.V., et al 2012
A10-0511	USA	Arizona	Flagstaff		*Urocyon cinereoargenteus*	2009	JQ685943	Cosmopolitan (AZFX)	Kuzmin,I.V., et al 2012
NC839	USA	Tennessee			*Mephitis mephitis*	1984	JQ685944	Cosmopolitan (NCSK)	Kuzmin,I.V., et al 2012
SM4862	USA	Arizona	Flagstaff		*Eptesicus fuscus*	1999	JQ685946	*Eptesicus fuscus*-W1	Kuzmin,I.V., et al 2012
TN310	USA	Tennessee			*Lasiurus cinereus*	2004	JQ685947	*Lasiurus cinereus*	Kuzmin,I.V., et al 2012
A093504	USA	Arizona	Flagstaff		*Eptesicus fuscus*	2001	JQ685950	*Eptesicus fuscus*-W1	Kuzmin,I.V., et al 2012
SM5101	USA	Arizona			*Mephitis mephitis*	2001	JQ685958	*Eptesicus fuscus*-W1	Kuzmin,I.V., et al 2012
SM4872	USA	Arizona	Flagstaff		*Eptesicus fuscus*	2001	JQ685960	*Eptesicus fuscus*-W1	Kuzmin,I.V., et al 2012
A10-0512	USA	Arizona			*Mephitis mephitis*	2009	JQ685968	South-central skunk	Kuzmin,I.V., et al 2012
CASK2	USA	California			*Mephitis mephitis*	1974	JQ685970	Cosmopolitan (CASK)	Kuzmin,I.V., et al 2012
AZ3003	USA	Arizona			*Antrozous pallidus*	2009	JQ685971	*Antrozous pallidus*	Kuzmin,I.V., et al 2012

Isolate	Country	State	County	City	Host source	Year	GenBank accession no.	RABV lineage	Reference
OR58	USA	Oregon			*Urocyon cinereoargenteus*	2010	JQ685977	*Eptesicus fuscus*-W2	Kuzmin,I.V., et al 2012
AW3565	USA	Alabama			*Lasionycteris noctivagans*	2010	JQ686001	*Lasionycteris noctivagans*	Kuzmin,I.V., et al 2012
A10-5102	USA	North Carolina			*Perimyotis subflavus*	2010	JQ686003	*Perimyotis subflavus*	Kuzmin,I.V., et al 2012
A11-5737	USA	Virginia			*Vulpes vulpes*	2011	JQ686009	*Perimyotis subflavus*	Kuzmin,I.V., et al 2012
ARDG090042	USA	Arkansas			*Canis familiaris*	2009	JX855994	Cosmopolitan (NCSK)	Davis,R., et al, 2013
SDSK090045	USA	South Dakota			*Mephitis mephitis*	2009	JX855997	Cosmopolitan (NCSK)	Davis,R., et al, 2013
ARSK090068	USA	Arkansas			*Mephitis mephitis*	2009	JX856015	South-central skunk	Davis,R., et al, 2013
OKBV090073	USA	Oklahoma			*Bos taurus*	2009	JX856017	South-central skunk	Davis,R., et al, 2013
NEBV090102	USA	Nebraska			*Bos taurus*	2009	JX856036	South-central skunk	Davis,R., et al, 2013
A08-1210	USA				*Lasionycteris noctivagans*	2008	JX871837	*Perimyotis subflavus*	Ellison,J.A., et al 2013
A10-2057	USA				*Eptesicus fuscus*	2010	JX871843	*Eptesicus fuscus*-W2	Ellison,J.A., et al 2013
A12-0276	USA				*Urocyon cinereoargenteus*	2012	KC792049	*Eptesicus fuscus*-W1	Kuzmina,N.A., et al 2013
A12-0331	USA				Bat	2012	KC792052	*Eptesicus fuscus*-W1	Kuzmina,N.A., et al 2013
A12-4122	USA				*Eptesicus fuscus*	2012	KC792129	*Eptesicus fuscus*-W1	Kuzmina,N.A., et al 2013
A12-6377	USA				Bat	2012	KC792145	*Lasionycteris noctivagans*	Kuzmina,N.A., et al 2013
A13-3207	USA				*Eptesicus fuscus*	2012	KJ174676	*Eptesicus fuscus*-W1	Kuzmina,N.A., et al, unpub. data
A13-4551	USA				*Urocyon cinereoargenteus*	2012	KJ174681	*Eptesicus fuscus*-W2	Kuzmina,N.A., et al, unpub. data
A13-4546	USA				*Myotis lucifugus*	2012	KJ174682	*Eptesicus fuscus* -W3	Kuzmina,N.A., et al., unpublish. data
A13-5626	USA				*Canis latrans*	2012	KJ174683	*Eptesicus fuscus* -W3	Kuzmina,N.A., et al, unpub. data
IP 350/10	Brazil				*Nyctinomops laticaudatus*	2010	KM594034	*Nyctinomops laticaudatus*	Oliveira, R.N. et al, 2014, unpub data
IP 542/10	Brazil				*Nyctinomops laticaudatus*	2010	KM594036	*Nyctinomops laticaudatus*	Oliveira, R.N. et al, 2014, unpub. data
05BC0708	Canada	Brackendale			*Myotis californicus*	2005	KY203100	*Myotis californicus*	Nadin-Davis,S.A., et al 2017
05BC0983	Canada	Summerland			*Myotis californicus*	2005	KY203103	*Myotis californicus*	Nadin-Davis,S.A., et al 2017
05ON2859	Canada				*Lasionycteris noctivagans*	2005	KY203107	*Lasionycteris noctivagans*	Nadin-Davis,S., et al, 2017
06ON0035	Canada				*Myotis leibii*	2006	KY203114	*Lasiurus seminolus*	Nadin-Davis,S., et al, 2017
RV50	USA				Bat	1975	MG458304	*Eptesicus fuscus*-W1	Fisher,S., et al 2018
A17-4052	USA	Oregon			Bat	2017	MW055098	*Eptesicus fuscus* - E2	Gigante,C.M., et al 2020
SHBRV	USA				*Lasionycteris noctivagans*	NA	U52946	*Lasionycteris noctivagans*	Morimoto,K., et al 1996

*Isolates not submitted to GenBank because of the length of the sequence was <200 bp. NA, not available; *numbers are not yet available.

References

1. Nadin-Davis SA, Huang W, Armstrong J, Casey GA, Bahloul C, Tordo N, et al. Antigenic and genetic divergence of rabies viruses from bat species indigenous to Canada. Virus Res. 2001;74:139–56. PubMed https://doi.org/10.1016/S0168-1702(00)00259-8

2. Rohde RE, Mayes BC, Smith JS, Neill SU. Bat rabies, Texas, 1996–2000. Emerg Infect Dis. 2004;10:948–52. PubMed https://doi.org/10.3201/eid1005.030719

3. Leslie MJ, Messenger S, Rohde RE, Smith J, Cheshier R, Hanlon C, et al. Bat-associated rabies virus in Skunks. Emerg Infect Dis. 2006;12:1274–7. PubMed https://doi.org/10.3201/eid1208.051526

4. Faber M, Pulmanausahakul R, Nagao K, Prosniak M, Rice AB, Koprowski H, et al. Identification of viral genomic elements responsible for rabies virus neuroinvasiveness. Proc Natl Acad Sci U S A. 2004;101:16328–32. PubMed https://doi.org/10.1073/pnas.0407289101

5. Shankar V, Orciari LA, De Mattos C, Kuzmin IV, Pape WJ, O'Shea TJ, et al. Genetic divergence of rabies viruses from bat species of Colorado, USA. Vector Borne Zoonotic Dis. 2005;5:330–41. PubMed https://doi.org/10.1089/vbz.2005.5.330

6. Biek R, Henderson JC, Waller LA, Rupprecht CE, Real LA. A high-resolution genetic signature of demographic and spatial expansion in epizootic rabies virus. Proc Natl Acad Sci U S A. 2007;104:7993–8. PubMed https://doi.org/10.1073/pnas.0700741104

7. Davis R, Nadin-Davis SA, Moore M, Hanlon C. Genetic characterization and phylogenetic analysis of skunk-associated rabies viruses in North America with special emphasis on the central plains. Virus Res. 2013;174:27–36. PubMed https://doi.org/10.1016/j.virusres.2013.02.008

8. Velasco-Villa A, Reeder SA, Orciari LA, Yager PA, Franka R, Blanton JD, et al. Enzootic rabies elimination from dogs and reemergence in wild terrestrial carnivores, United States. Emerg Infect Dis. 2008;14:1849–54. PubMed https://doi.org/10.3201/eid1412.080876

9. Streicker DG, Turmelle AS, Vonhof MJ, Kuzmin IV, McCracken GF, Rupprecht CE. Host phylogeny constrains cross-species emergence and establishment of rabies virus in bats. Science. 2010;329:676–9. PubMed https://doi.org/10.1126/science.1188836

10. Aréchiga-Ceballos N, Velasco-Villa A, Shi M, Flores-Chávez S, Barrón B, Cuevas-Domínguez E, et al. New rabies virus variant found during an epizootic in white-nosed coatis from the Yucatan Peninsula. Epidemiol Infect. 2010;138:1586–9. PubMed https://doi.org/10.1017/S0950268810000762

11. Caicedo Y, Paez A, Kuzmin I, Niezgoda M, Orciari LA, Yager PA, et al. Virology, immunology and pathology of human rabies during treatment. Pediatr Infect Dis J. 2015;34:520–8. PubMed https://doi.org/10.1097/INF.0000000000000624

12. Streicker DG, Altizer SM, Velasco-Villa A, Rupprecht CE. Variable evolutionary routes to host establishment across repeated rabies virus host shifts among bats. Proc Natl Acad Sci U S A. 2012;109:19715–20. PubMed https://doi.org/10.1073/pnas.1203456109

13. Kuzmin IV, Shi M, Orciari LA, Yager PA, Velasco-Villa A, Kuzmina NA, et al. Molecular inferences suggest multiple host shifts of rabies viruses from bats to mesocarnivores in Arizona during 2001–2009. PLoS Pathog. 2012;8:e1002786. PubMed https://doi.org/10.1371/journal.ppat.1002786

14. Ellison JA, Johnson SR, Kuzmina N, Gilbert A, Carson WC, VerCauteren KC, et al. Multidisciplinary approach to epizootiology and pathogenesis of bat rabies viruses in the United States. Zoonoses Public Health. 2013;60:46–57. PubMed https://doi.org/10.1111/zph.12019

15. Kuzima NA, Kuzmin IV, Ellison JA, Rupprecht CE. Conservation of binding epitopes for monoclonal antibodies on the rabies virus glycoprotein. J Antivir Antiretrovir. 2013;5:2.

16. Oliveira RN, Freire CC, Iamarino A, Zanotto PM, Pessoa R, Sanabani SS, et al. Rabies virus diversification in aerial and terrestrial mammals. Genet Mol Biol. 2020;43:e20190370–20190370. PubMed https://doi.org/10.1590/1678-4685-gmb-2019-0370

17. Nadin-Davis S, Alnabelseya N, Knowles MK. The phylogeography of *Myotis* bat–associated rabies viruses across Canada. PLoS Negl Trop Dis. 2017;11:e0005541. PubMed https://doi.org/10.1371/journal.pntd.0005541

18. Fischer S, Freuling CM, Müller T, Pfaff F, Bodenhofer U, Höper D, et al. Defining objective clusters for rabies virus sequences using affinity propagation clustering. PLoS Negl Trop Dis. 2018;12:e0006182. PubMed https://doi.org/10.1371/journal.pntd.0006182

19. Gigante CM, Yale G, Condori RE, Costa NC, Long NV, Minh PQ, et al. Portable rabies virus sequencing in canine rabies endemic countries using the Oxford Nanopore MinION. Viruses. 2020;12:1255. PubMed https://doi.org/10.3390/v12111255

20. Morimoto K, Patel M, Corisdeo S, Hooper DC, Fu ZF, Rupprecht CE, et al. Characterization of a unique variant of bat rabies virus responsible for newly emerging human cases in North America. Proc Natl Acad Sci U S A. 1996;93:5653–8. PubMed https://doi.org/10.1073/pnas.93.11.5653

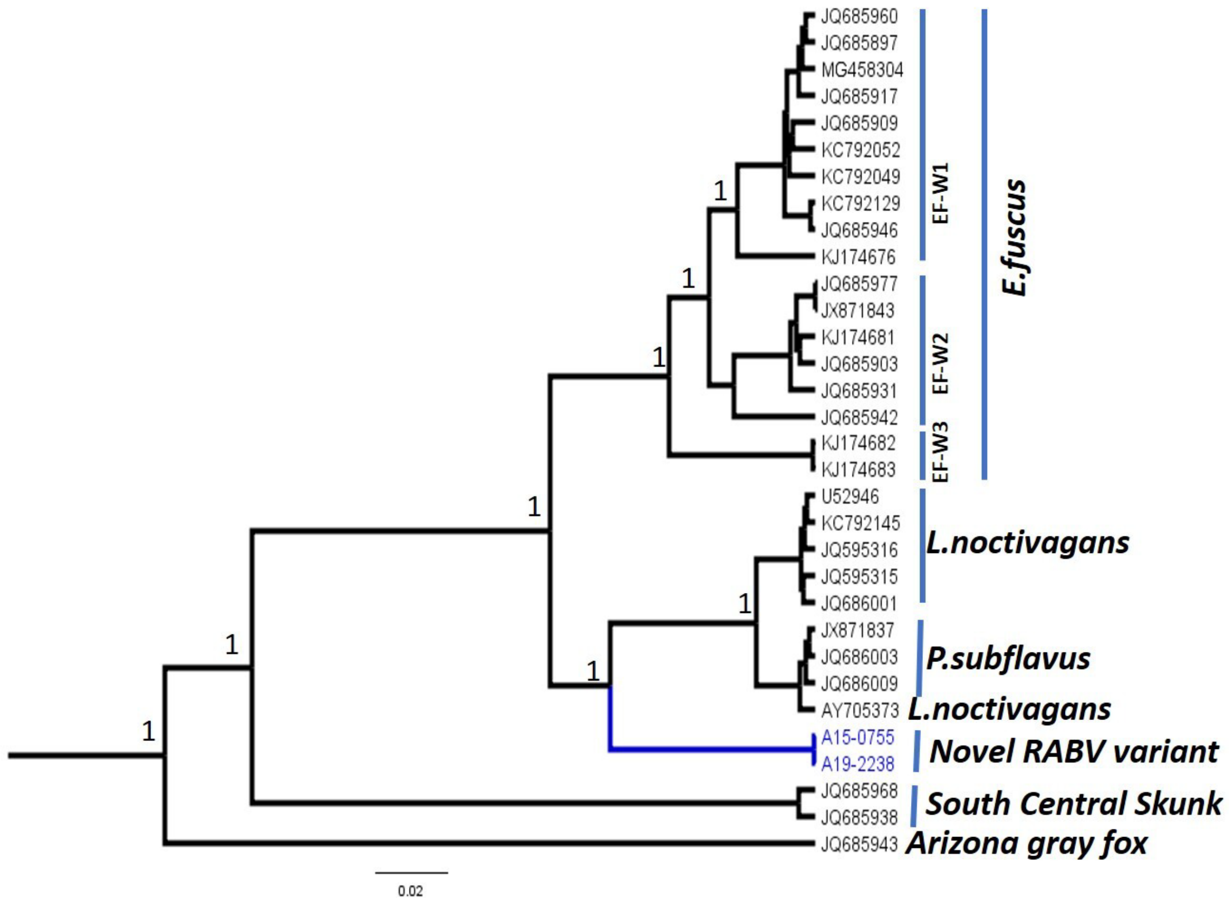

Appendix 2 Figure. Maximum clade credibility tree using full glycoprotein gene sequences of the novel rabies virus variant in blue and representative sequences of RABV associated with terrestrial mammals and bats (*Lasionycteris noctivagans*, *Perimyotis subflavus*, and *Eptesicus fuscus*). RABV, rabies virus.
